# Inhibition of *Salmonella* Typhimurium adhesion, invasion, and intracellular survival via treatment with methyl gallate alone and in combination with marbofloxacin

**DOI:** 10.1186/s13567-018-0597-8

**Published:** 2018-10-04

**Authors:** Biruk Tesfaye Birhanu, Na-Hye Park, Seung-Jin Lee, Md Akil Hossain, Seung-Chun Park

**Affiliations:** 1 Laboratory of Veterinary Pharmacokinetics and Pharmacodynamics, College of Veterinary Medicine, Kyungpook National University, Bukgu, Daegu, 41566 South Korea; 2 Veterinary Drugs and Biologics Division, Animal and Plant Quarantine Agency, Gimcheon, 39660 South Korea

## Abstract

*Salmonella enterica* serovar Typhimurium infects intestinal epithelia and macrophages, which is prevented by inhibiting adhesion and cell invasion. This study aimed to investigate the role of methyl gallate (MG) in adhesion, invasion, and intracellular survival of *Salmonella* Typhimurium in Caco-2 and RAW 264.7 cells via a gentamicin protection assay, confocal microscopy, and quantitative reverse-transcription polymerase chain reaction. MG (30 µg/mL) inhibited adhesion and invasion of *Salmonella* Typhimurium by 54.01% and 60.5% in RAW 264.7 cells, respectively. The combination of MG with sub-minimum inhibitory concentration (MIC) of marbofloxacin (MRB) inhibited the adhesion, invasion, and intracellular survival by 70.49%, 67.36%, and 74%, respectively. Confocal microscopy further revealed reductions in bacterial count in Caco-2 cells treated with MG alone or with sub-MIC of MRB. Furthermore, MG alone or in combination with sub-MIC of MRB decreased the motility of *Salmonella* Typhimurium. Quorum sensing genes including *sdiA*, *srgE*, and *rck* were downregulated by 52.8%, 61.7%, and 22.2%, respectively. Moreover, *rac*-*1* was downregulated by 56.9% and 71.9% for MG alone and combined with sub-MIC of MRB, respectively, in mammalian cells. Furthermore, MG downregulated virulence genes of *Salmonella* Typhimurium including *cheY, ompD*, *sipB*, *lexA*, and *ompF* by 59.6%, 60.2%, 20.5%, 31.4%, and 16.2%, respectively. Together, the present results indicate that MG alone or in combination with a sub-MIC of MRB effectively inhibited the adhesion, invasion, and intracellular survival of *Salmonella* Typhimurium in vitro by downregulating quorum sensing and virulence genes.

**Electronic supplementary material:**

The online version of this article (10.1186/s13567-018-0597-8) contains supplementary material, which is available to authorized users.

## Introduction

*Salmonella enterica* serovar Typhimurium is a gram-negative facultative anaerobic enteric pathogen in humans and animals, and a leading cause of gastroenteritis [1]. The strain invades intestinal phagocytic and epithelial (nonphagocytic) cells. Bacterial adhesion is crucial to cause an infection, enabling the persistence of extracellular bacteria in the host and resulting in the internalization of intracellular bacteria within host cells [2]. Hence, the entry of *S. enterica* into epithelial cells is important for its pathogenicity, intracellular replication, dissemination to other tissues, and establishment of intestinal diseases [3–6].

*Salmonella* strains penetrate nonphagocytic cells via a trigger or zipper mechanism. The *Salmonella* pathogenicity island-1 (SPI-1) type III secretion system (T3SS) is critical for invasion of host cells via the trigger mechanism by deploying a macropinocytosis-related process in enterocytes and the SPI-2 of the T3SS is responsible for the zipper mechanism and intracellular survival of *Salmonella* Typhimurium [7, 8]. Effector proteins of *Salmonella* SPI-1 regulate cellular invasion and enable the rearrangement of the actin cytoskeleton in the host cell. These proteins indirectly regulate the activation of the Rho GTPases including the CDC42 and Rac1 proteins in host cells [9, 10]. However, Rck alone can regulate the adhesion and cellular penetration of *Salmonella* strains via the zipper mechanism [7].

Inhibition of bacterial adhesion, invasion, and intracellular survival significantly limits the pathogenicity of microbial agents and for the prevention and control of infections [11]. Various antimicrobial agents are used to treat intracellular bacterial infections in humans and animals [12]. Fluoroquinolones are among the most common antibiotics used to treat gastroenteritis and are used primarily against multi-drug resistant microbial agents. However, bacteria have developed resistance against these antibiotics. In addition, certain antibiotics at their inhibitory concentration failed to eliminate intracellular surviving bacteria [13].

Ciprofloxacin cannot eliminate intracellular *Salmonella* even at a higher dosage [14]. Similarly, enrofloxacin, used in veterinary medicine, remained ineffective against intracellular *Salmonella*. Furthermore, this bacterium has developed resistance to various fluoroquinolones including marbofloxacin (MRB), which is a widely used antibacterial agent in veterinary medicine to treat digestive and respiratory tract infections [15]. This may lead to treatment failure, increased antimicrobial resistance, and increased occurrence of drug failure and cytotoxicity due to frequent, high doses [16, 17]. To overcome these issues, a combination of fluoroquinolone with natural products can serve as a novel therapeutic strategy [13].

Certain naturally occurring phenolic compounds have antioxidant, anticarcinogenic, and antimicrobial activity [18, 19]. Methyl gallate (MG), a polyphenolic compound with three hydroxyl rings, has anti-inflammatory, antioxidant, anticancer, antiviral, anti-asthmatic, and vasodilating activities [18]. Recently, we reported that MG regulated bacterial quorum sensing (QS) pathways [20]. QS signaling plays a significant role in bacterial metabolism, increase bacterial pathogenicity, and antimicrobial resistance [21]. However, the role of MG in inhibiting bacterial adhesion, invasion, and intracellular survival is unknown.

Hence, we hypothesize that MG reduces the virulence of *Salmonella* Typhimurium and contributes to the reduction of antibacterial resistance by inhibiting QS signaling when administered alone or in combination with MRB. Therefore, in this study, we investigated the effects and mechanism of the inhibition of *Salmonella* Typhimurium adhesion, invasion, and intracellular survival in cell cultures via treatment with MG alone and in combination with sub-inhibitory concentration (sub-MIC) of MRB to combat drug-resistance via downregulation of genes involved in QS signaling.

## Materials and methods

### Chemicals, antimicrobials, and reagents

All chemicals, reagents, and antibiotics used in the experiments were procured from Sigma-Aldrich (Sigma, St. Louis, MO, USA) unless otherwise specified.

### Bacteria and cell culture

Three *Salmonella enterica* subspecies *enterica* serovar Typhimurium strains were used. Two field isolates from swine clinical infections, wherein one was susceptible (0.031 µg/mL), while the other was resistant (0.5 µg/mL) to MRB [15]. In addition, ATCC 14028 was used as a control. All the bacteria were grown in Luria–Bertani (LB) broth (Difco, BD, Sparks, MD, USA) at 37 °C overnight. For the invasion assay, bacteria were cultured in LB broth supplemented with 0.3 M NaCl in a non-shaking incubator at 37 °C to induce gene expression. Two cell lines, a macrophage RAW 264.7 cell line and an epithelial Caco-2 cell line, were used for cell culture experiments. RAW 264.7 cells were cultured in RPMI 1640 medium supplemented with 10% fetal bovine serum (FBS) and 1% penicillin–streptomycin (P/S). Caco-2 cells were cultured in minimum essential medium (MEM, Gibco, Grand Island, NY, USA) supplemented with 1% non-essential amino acids, 20% FBS, and 1% P/S. All cell cultures were incubated at 37 °C and 5% CO_2_.

### Cell viability assay

The effect of MG on the survival rate of the host cell was determined via a 3-(4,5-dimethyl-2-thiazolyl)-2,5-diphenyl-2H-tetrazolium bromide (MTT) assay. Confluent RAW 264.1 cells were seeded in a 96-well plate at a density of 10^5^ cells/mL and incubated for 24 h at 37 °C and 5% CO_2_. The medium was aspirated and fresh medium containing a twofold dilution of MG, starting from 2000 μg/mL, was added before being incubated overnight at 37 °C and 5% CO_2_. The medium was substituted with fresh medium containing 0.45 mg/mL of MTT reagent and incubated for 4 h. Finally, dimethyl sulfoxide was added, and the optical density was measured after 5 min at 570 nm, using a VersaMax^®^ microplate reader (Molecular Devices, Sunnyvale, CA, USA). The test was performed thrice in duplicate. The survival rate was calculated using the following formula:

$${\text{Survival rate }} = \frac{(OD\, extract - OD \,blank)}{(OD\, control\, cells - OD\, blank)} \times 100$$

### Nitric oxide assay

The nitric oxide (NO) inhibition test was conducted using RAW 264.7 cells (10^5^ cells/mL) in 24-well plates with Griess reagents to confirm the anti-inflammatory activity of MG. MG at different concentrations (300, 100, 30, and 10 µg/mL) was added to the cells 30 min before inoculation with *Salmonella* Typhimurium (10^7^ CFU/mL) to make the multiplicity of infection (MOI) 1:100. These mixtures were incubated for 24 h at 37 °C and 5% CO_2_. The test was conducted using a 96-well plate and twofold serially diluted standard NO starting with 100 µM and intact *Salmonella* and lipopolysaccharide (LPS, 1 µg/mL) as the positive control; negative control, untreated cells. The test was conducted three times in duplicate and the optical density was measured at 540 nm, using a VersaMax^®^ microplate reader.

### Invasion, adhesion, and intracellular killing assay

The effect of MG alone and in combination with MRB on bacterial invasion was determined using the gentamicin protection assay, as described previously [22], with some modifications. Briefly, MG (30 µg/mL) alone or with sub-MIC of MRB (0.015 µg/mL for susceptible and 0.25 µg/mL for resistant bacteria) was added to fully confluent Caco-2 cells (10^5^ cells/mL) and incubated for 30 min. *Salmonella* Typhimurium (10^7^ CFU/mL) were added and incubated for another 45 min after being centrifuged at 500 × *g* for 5 min. Gentamicin (100 µg/mL) was added and incubated for 30 min before cells were lysed with 0.1% Triton^®^ x-100 for 10 min. Finally, the suspension was serially diluted and cultured on LB agar plates for viable counting of bacteria after overnight incubation. The same procedure was followed for the adhesion inhibition assay, except for treatment with gentamicin. The experiments were performed at least eight times in duplicate.

Similarly, for the intracellular killing experiment, RAW 264.7 cells (10^5^ cells/mL) were cultured in 24-well plates. The cells were treated with *Salmonella* Typhimurium (10^7^ CFU/mL) and incubated for 45 min after centrifugation at 500 *g* for 5 min. After the cells were washed, MG (30 µg/mL) alone or with sub-MIC of MRB was added and incubated for 1 h at 37 °C and 5% CO_2_. Finally, cells were treated with gentamicin (100 µg/mL) for 1 h and lysed with 0.1% of Triton × 100 before being serially diluted and plated on LB agar.

For all experiments, cells infected with *Salmonella* Typhimurium without treatment and those treated with sub-MIC of MRB were used as the control.

### Confocal microscopy

Confocal microscopy was performed to visualize and compare the extent of bacterial invasion after treatment with MG. Caco-2 cells (10^5^ cells/mL) were cultured on 12-mm glass coverslips in 24-well plates, as described previously [23]. Cells were prepared and treated as described above. Cells were gently rinsed once in 0.1 M 3-(*N*-morpholino) propanesulfonic acid (MOPS), pH 7.2, containing 1 mM MgCl_2_ (MOPS/MgCl_2_). The rinsing solution from the cells was aspirated and 0.5 mL Live/Dead Staining Solution containing 5 μM SYTO9, 30 μM propidium iodide (Invitrogen, Thermo Fisher Scientific, Eugene, OR, USA), and 0.1% saponin in MOPS/MgCl_2_ was added. The cells were incubated for 15 min in the dark at room temperature before rinsing in MOPS/MgCl_2_. The coverslips were inverted face down onto glass slides and sealed with clear nail polish. Images were acquired within 30 min, using a Carl Zeiss (LSM700) confocal microscopy.

### Motility assay

Since bacterial motility is critical for its virulence, we sought to determine the effect of MG alone and in combination with sub-MIC of MRB on the motility of *Salmonella* Typhimurium. The motility assay was conducted in LB medium containing 0.3% (W/V) agar as previously described [24]. Briefly, 5 µL of an 8 h culture of *Salmonella* Typhimurium was inoculated in medium containing sub-MIC of MRB or MG at 30 or 100 µg/mL, respectively, or its combination with sub-MIC of MRB, using medium without treatment as a control. The plates were incubated at 37 °C for 12 h, and the diameter of bacterial spread was measured using calipers.

### Assessment of the expression of genes involved in quorum sensing

A quorum sensing signaling autoinducer-1 (AI-1) has not been described for *Salmonella* Typhimurium [25]. Hence, to determine the effect of MG on the expression of QS genes of *Salmonella* Typhimurium [26], *N*-acyl homoserine lactone (AHL) (1 μmol/mL) was added to LB broth containing bacteria (10^7^ CFU/mL) and incubated with 30 or 100 μg/mL of MG alone or in combination with sub-MIC of MRB for 8 h. After centrifugation at 13 000 × *g* for 5 min at 4 °C the pellet was processed for RNA extraction. Total RNA was extracted using Trizol^®^ (Ambion^®^, Life Technologies, Carlsbad, CA, USA) reagent in accordance with the manufacturer’s instructions. RNA purity and concentration were measured using Nanophotometer (Implen GmbH, Munich, Germany). A quantitative reverse transcription-PCR (qRT-PCR) pre-mix (Pioneer, Korea) was used to synthesize cDNA by adding random hexamers to the bacterial RNA. RNA was quantified using a CFX96 Touch™ real-time PCR detection system (Bio-Rad, Singapore) using IQ™ SYBR^®^ Green supermix for real time PCR (Bio Rad, Singapore). Gene expression levels of *sdiA, rsgE*, and *rck* were determined via quantitative reverse transcription polymerase chain reaction (qRT-PCR) Under the following cycling conditions: 95 °C for 30 s, followed by 40 cycles at 95 °C for 5 s, 60 °C for 30 s, and dissociation at 95 °C for 15 s, followed by 60 °C for 30 s. Target gene expression levels were normalized to that of housekeeping gene *rrsG* in *Salmonella* Typhimurium, using the 2^−ΔΔCT^ method.

### Gene expression in host cells

The effect of MG on host gene expression after invasion by *Salmonella* Typhimurium was also determined via qRT-PCR. Cells were infected with *Salmonella* Typhimurium, as described for the invasion assay, and total RNA was extracted using Trizol^®^ reagent as described above. cDNA was synthesized using oligo (dT) primers. The conditions for qRT-PCR was set for 95 °C for 7 min and 30 cycles of 95 °C for 30 s, 58 °C for 30 s and 72 °C for 1 min. Target gene expression levels were normalized to that of β-actin (housekeeping gene; internal control), using the 2^−ΔΔCT^ method.

### Virulence gene expression and total RNA extraction

To assess virulence gene expression, *Salmonella* Typhimurium (10^7^ CFU/mL) was incubated in LB broth containing 0.3 M of NaCl and MG and MRB at different concentrations at 37 °C for 13 h without agitation. Total RNA was extracted as described above. qRT-PCR for *sipB, ompD*, *ompF*, *cheY*, *lexA* and *rck* genes was performed under the following cycling conditions: 95 °C for 5 min, 40 cycles at 95 °C for 30 s, 60 °C for 30 s, 72 °C for 1 min and final annealing and extension of 55 °C for 5 s and 95 °C for 30 s, respectively. The primers used in the study are listed in Additional file 1.

### Statistical analysis

Graphpad prism 7 (GraphPad Software, La Jolla, CA, USA) was used to analyze the results. One-way and two-way analysis of variance (ANOVA) followed by using Tukey’s multiple comparison test were used to compare means among the treatment groups and to compute the P-value. Statistical significance was set at *P* < 0.05.

## Results

**Figure 1:**
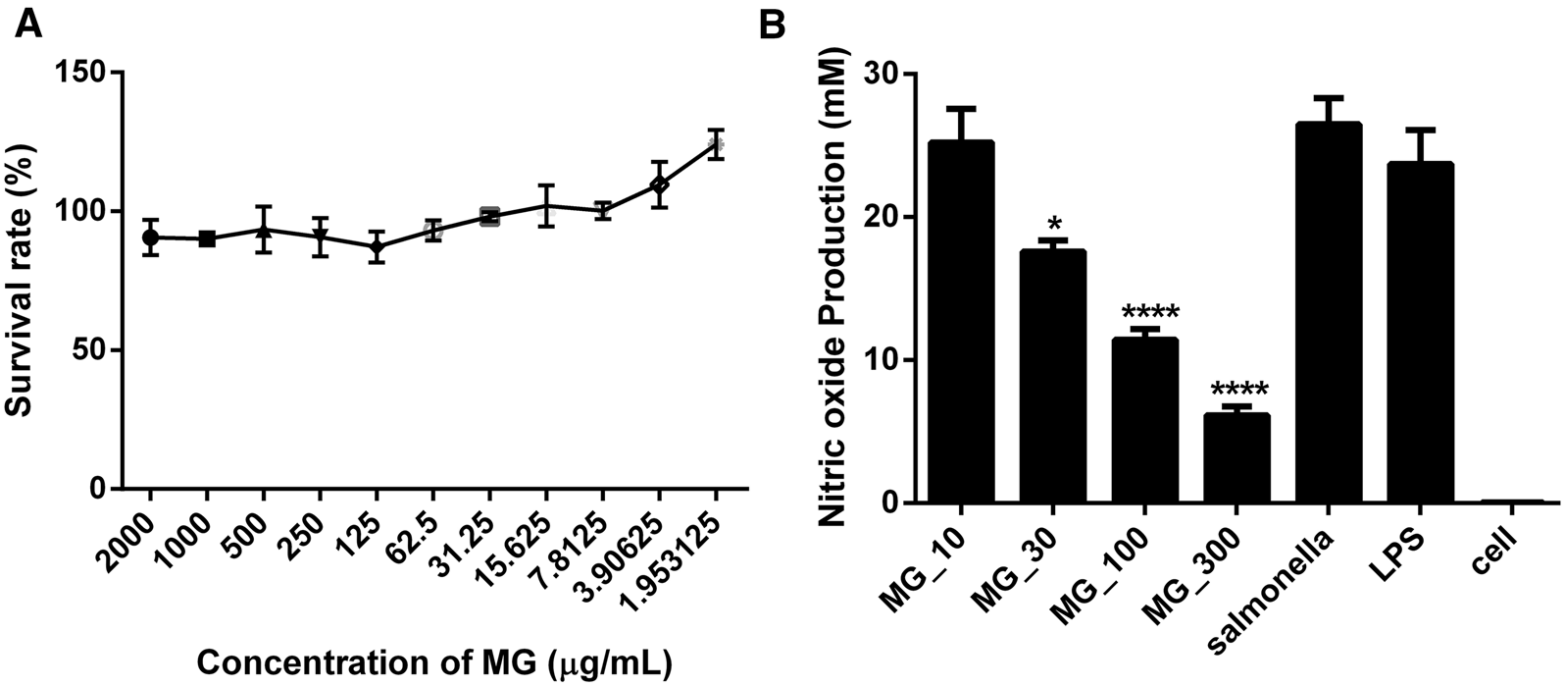
**Effect of methyl gallate on cell survival and nitric oxide production in** ***Salmonella*** **Typhimurium (ATCC 14028). A** Effect of methyl gallate on cell survival, **B** methyl gallate inhibited nitric oxide in showing a dose-dependent manner in intact *Salmonella* Typhimurium in Raw 264.7 cells after 24 h of incubation. Values indicated are the mean ± SEM (*n* = 6; *P-value < 0.05; ***P-value < 0.001).

The cytotoxic effect of MG on mammalian cells was determined using Caco-2 and Raw 264.7 cell lines. MG, at its highest concentration (2 mg/mL), decreased the survival rate of the cells only by 9.5%. The survival rate was increased to 93%, at 62.5 µg/mL. Hence, afterwards we used a concentration of 30 µg/mL, which yielded a survival rate of 98% (Figure 1A).

To determine the effect of MG on NO produced by RAW 264.7 cells induced by intact *Salmonella* Typhimurium, Griess reagent was used. MG displayed dose-dependent inhibition of NO induced by the *Salmonella* Typhimurium strain ATCC 14028 on RAW 264.7 cells. NO levels were reduced by 4.8, 33.6, 57, and 76.8% for cells treated with 10, 30, 100, and 300 µg/mL of MG after 24 h of incubation, respectively (Figure 1B). A significant difference was observed among cells treated with 30, 100, and 300 µg/mL of MG in comparison with nontreated cells.

The minimum inhibitory concentration (MIC) of MG was determined using the broth micro-dilution method on cation-adjusted Muller Hinton broth (Difco) and RPMI media. After making two-fold dilutions of MG, bacteria at a final density of 10^5^ CFU/mL was added to the 96-well plate and incubated for 24 h at 37 °C. For the three strains tested, we obtained an MIC of 500 µg/mL and at less than 125 µg/mL, no bacterial inhibition was observed. Hence, afterwards we used a lower concentration of MG (30 µg/mL), which does not have any antibacterial activity.

**Figure 2:**
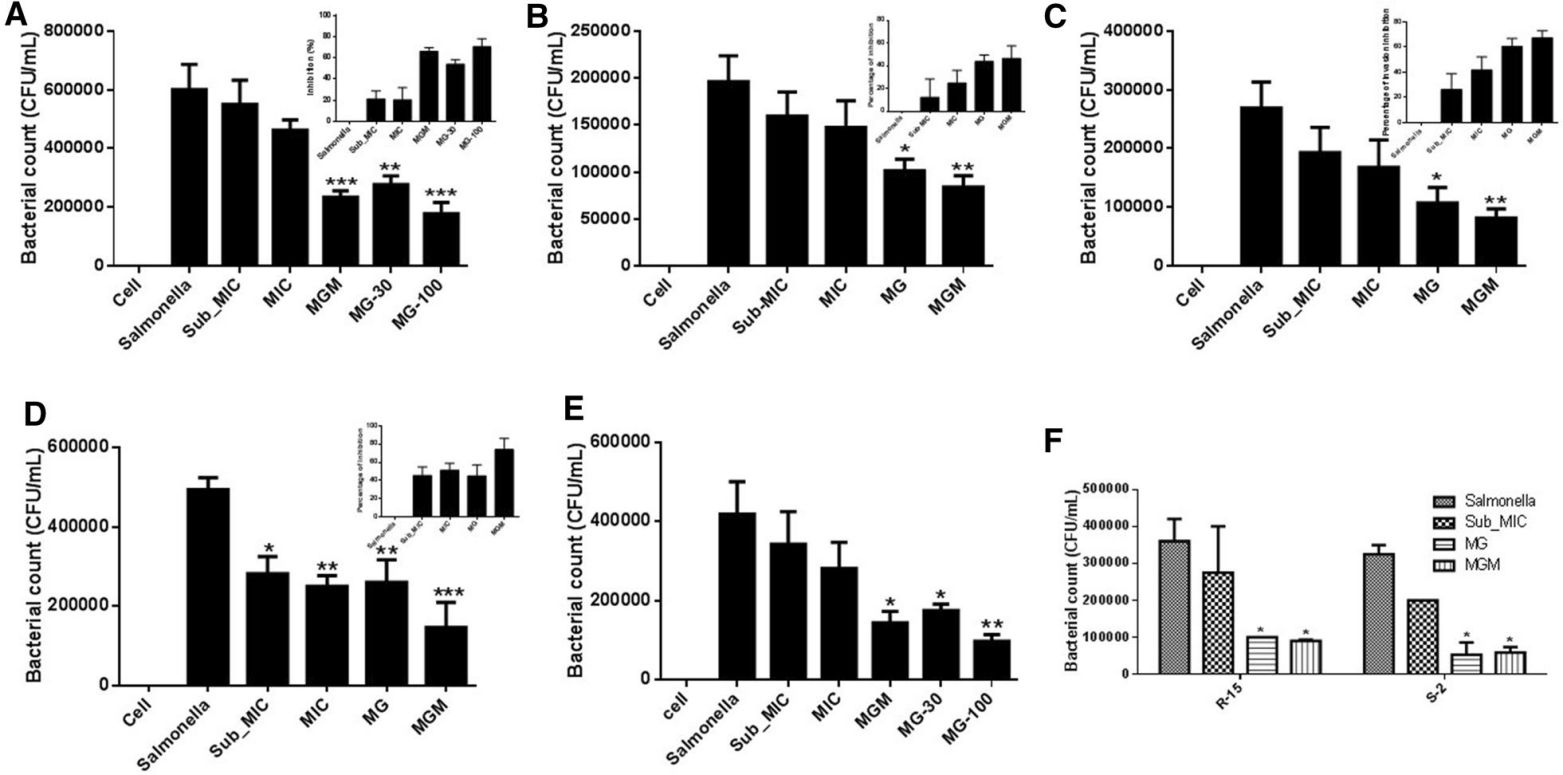
**Inhibitory effects of methyl gallate (MG) on adhesion, invasion, and intracellular survival of** ***Salmonella*** **Typhimurium alone and in combination with a sub-minimum inhibitory concentration (MIC) of marbofloxacin (MRB). A** Effect of MG alone and in combination with sub-MIC of MRB on adhesion of *Salmonella* Typhimurium; **B** effect of MG alone and in combination with sub-MIC of MRB on the invasion of *Salmonella* Typhimurium on Caco-2 cells; **C** invasion of Raw 264.7 cells, **D** effect of MG alone and in combination with sub-MIC of MRB on the intracellular survival of *Salmonella* Typhimurium in Raw 264.7 cells; **E** dose-dependent inhibition of invasion by MG; **F** inhibition of invasion by MG on two different field isolates of *Salmonella* Typhimurium. The upper figures show the percentage of inhibition. Cell, uninfected and non-treated control; *Salmonella,* infected with *Salmonella* Typhimurium but left untreated; Sub-MIC and MIC, controls treated with the sub-MIC and MIC of MRB; MG-30, treated with 30 µg/mL of methyl gallate alone; MGM, treatment with a combination of methyl gallate (30 µg/mL) and sub-MIC of MRB; MG-100, treated with 100 µg/mL of methyl gallate alone. Results represent the mean ± SEM (*n* = 8; *P-value < 0.05; **P-value < 0.01; ***P-value < 0.001).

Inhibition of bacterial adhesion, invasion, and intracellular survival was assessed using a gentamicin protection assay. MG showed inhibitory activity against adhesion of *Salmonella* Typhimurium. Adhesion was inhibited by 54.01 and 70.49% by MG at a concentration of 30 and 100 µg/mL, respectively. The combination of sub-MIC of MRB (0.015 µg/mL) and MG (30 µg/mL), however, inhibited 66% of bacterial adhesion (Figure 2A), whereas, the MIC and sub-MIC of MRB alone inhibited 20.4 and 21.2%, respectively. The inhibition of adhesion by the combination treatment was significantly (*P* < 0.0001) greater than that in the non-treated control group, in a dose-dependent manner.

Similarly, in the invasion assay, MG inhibited 43.75 and 46.4% of *Salmonella* Typhimurium at 30 µg/mL alone and in combination with the sub-MIC (0.015 µg/mL) of MRB, respectively in Caco-2 cells. Whereas, the inhibition of invasion by the MIC and sub-MIC of MRB alone was 24.8 and 12.2%, respectively when compared to the non-treated *Salmonella* Typhimurium. The inhibition increased to 60.5 and 67.36% in Raw 264.7 cells on using MG alone and in combination with sub-MIC of MRB, respectively (Figures 2B and C). While, the MIC and sub-MIC of MRB inhibited the invasion of the bacteria in RAW 264.7 cells by only 41.9 and 26.4%, respectively. A significant difference (*P* < 0.0001) was observed in the inhibition of *Salmonella* Typhimurium invasion by MG alone and its combination with sub-MIC of MRB. However, no significant difference was observed between the assay carried out using Caco-2 and Raw 264.7 cell lines.

MG inhibited invasion in a dose-dependent manner (Figure 2E). Furthermore, no significant difference was observed between the tested ATCC 14028 and two field (resistant and susceptible to MRB) isolates of *Salmonella* Typhimurium (Figure 2F).

MG also decreased the intracellular-survival of *Salmonella* Typhimurium alone and in combination with the sub-MIC of MRB. MG decreased the intracellular survival of *Salmonella* Typhimurium by 45% and it suppressed 74% of the infecting *Salmonella* Typhimurium in combination with sub-MIC of MRB (Figure 2D); this difference was significant (*P* < 0.0001). The intracellular survival suppression by the MIC and sub-MIC of MRB was 51.3 and 45.6%, respectively compared with the non-treated *Salmonella* Typhimurium.

**Figure 3:**
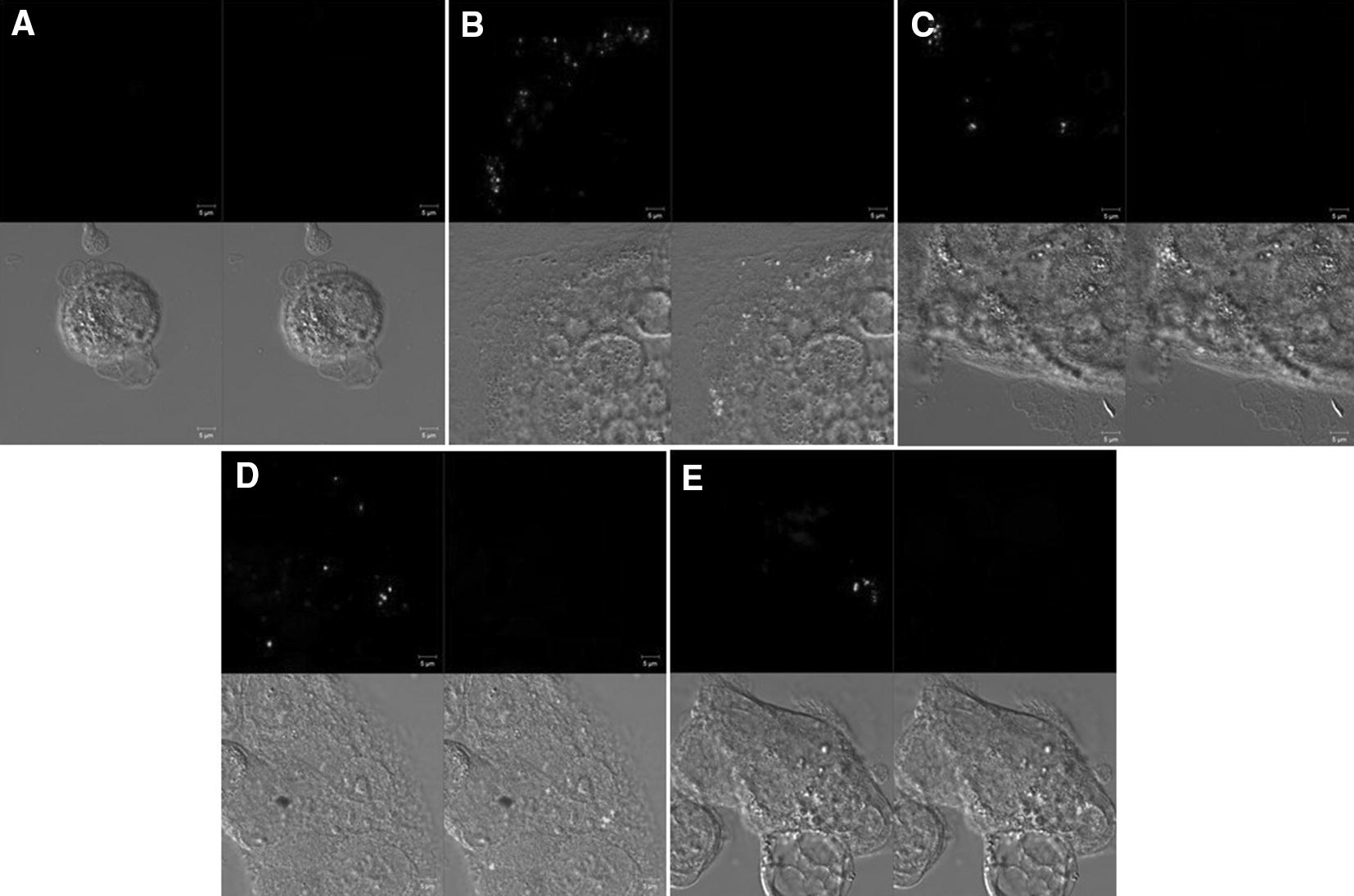
**Confocal microscopy of Caco-2 cells infected with** ***Salmonella*** **Typhimurium. A** Un-infected cell; **B** infected with *Salmonella* Typhimurium but not treated; **C** infected with *Salmonella* Typhimurium and treated with sub-MIC of MRB; **D** infected with *Salmonella* Typhimurium and treated with MG (30 µg/mL) alone; **E** infected with *Salmonella* Typhimurium and treated with a combination of methyl gallate (30 µg/mL) and sub-MIC of MRB. Upper left panel—SYTO-9; upper right panel—PI; lower left panel—cell structure, and lower right panel show merged images. (Red, dead bacteria; Green, Live bacteria; image: ×1000 magnification; representative images of three different experiments are presented).

Invading *Salmonella* Typhimurium in the host cells was confirmed via confocal microscopy. The intracellular bacteria were stained with SYTO-9 and propidium iodide to visualize live and dead cells, respectively. Concurrently, confocal microscopy revealed a reduction in the number of live invading bacteria after treatment with MG alone and in combination with sub-MIC of MRB (Figure 3).

**Figure 4:**
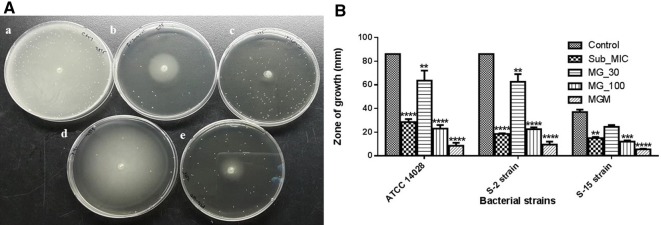
**Inhibition of** ***Salmonella*** **Typhimurium motility by methyl gallate alone and in combination with a sub-minimum inhibitory concentration (MIC) of marbofloxacin**. **A** Inhibition of *Salmonella* motility on semi-solid LB plate, bacteria were inoculated at the center of the LB plate containing 0.3% agar and incubated for 13 h at 37 °C. (a) *Salmonella* Typhimurium inoculated in LB plate containing no antibiotic (b) plate containing sub-MIC of MRB and inoculated with *Salmonella* Typhimurium, (c) plate containing the combination of MG (30 μg/mL) with sub-MIC of MRB and inoculated with *Salmonella* Typhimurium, (d) plate containing 30 µg/mL of MG and inoculated with *Salmonella* Typhimurium, and (e) plate containing 100 µg/mL of MG and inoculated with *Salmonella* Typhimurium. **B** Inhibition of *Salmonella* Typhimurium motility in three different strains by MG. Images are representatives of three different experiments. The control comprises *Salmonella* Typhimurium cultured on agar plates without any drugs; sub-MIC refers agar plate containing sub-MIC of MRB; MG-30 refers to treated with 30 µg/mL of methyl gallate alone; MGM refers to the treatment with a combination of methyl gallate (30 µg/mL) and sub-MIC of MRB; MG-100 refers to treated with 100 µg/mL of methyl gallate.

As shown in Figure 4, MG inhibited the motility of *Salmonella* Typhimurium in all three strains. The zone of *Salmonella* Typhimurium motility treated with sub-MIC of MRB was 28.5, 19.0, and 14.0 mm for the ATCC 14028, field susceptible strain (S-2), and resistant strain (R-15), respectively. MG at 30 µg/mL yielded a zone of inhibition of 63.5, 69.0, and 23.0 mm, and at 100 µg/mL, the zone was reduced to 23, 21, and 11 mm against the ATCC 14028 strain, S-2 and R-15 strains, respectively. However, higher inhibition of motility was observed when MG (30 µg/mL) was combined with sub-MIC of MRB and the growth zone was 8.5, 7, and 5 mm, for the ATCC 14028, S-2, and R-15, respectively. Whereas, the non-treated *Salmonella* Typhimurium showed a full growth on 90 mm petri dish (Figure 4).

**Figure 5:**
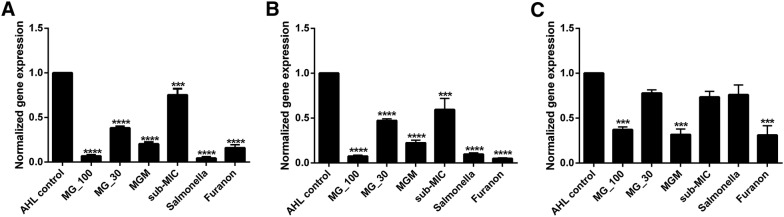
**Effect of methyl gallate (MG) on the expression of** ***Salmonella*** **Typhimurium quorum sensing genes (*sdiA***, ***srgE*, and** ***rck*). A** Effect of MG on *srgE* expression, **B** effect of MG on *sdiA* expression, and **C** effect of MG on *rck* expression. The genes were activated by 1 µmol of *N*-acetyl homoserine lactone (AHL) and treated with 100 and 30 µg/mL of MG and its combination with a sub-minimum inhibitory concentration (MIC) of MRB before incubation for 8 h. AHL-control, inoculated with *Salmonella* Typhimurium in the presence of AHL; MG_100, inoculated with *Salmonella* Typhimurium in the presence of AHL and 100 µg/mL of NG; MG_30, inoculated with *Salmonella* Typhimurium in the presence of AHL and 30 µg/mL of MG; MGM, inoculated with *Salmonella* Typhimurium in the presence of AHL and a combination of sub-MIC of MRB with 30 µg/mL of MG; sub-MIC, inoculated with *Salmonella* Typhimurium in the presence of AHL and sub-MIC of MRB; Salmonella, inoculated with *Salmonella* Typhimurium without AHL; Furanon, inoculated with *Salmonella* Typhimurium in the presence of AHL and 10 mg/mL of furanone as a positive control. Values indicated are the mean ± SEM (*n* = 3; ***P-value < 0.001).

MG affects QS signaling in bacteria [16]. However, no studies have investigated this inhibition in *Salmonella* Typhimurium. Since *Salmonella* Typhimurium has no QS signaling molecule, we treated the bacteria by adding AHL (1 µM/mL) in the LB broth to induce QS signals and determined the effect of MG on the expression of two QS genes of *Salmonella* Typhimurium, *sdiA* and *srgE*. Furanone (10 µg/mL) was used as a positive control. Treatment of *Salmonella* Typhimurium with 30 µg/mL of MG downregulated *sdiA* by 52.8%, which was increased to 92.6 and 77.7% for 100 µg/mL of MG and its combination with sub-MMIC of MRB, respectively, in the presence of AHL. Whereas, the suppression by the sub-MIC of MRB was 40.6%. *srgE* was downregulated by 24.7, 61.7, 93.2, and 79.5% for *Salmonella* treated with only sub-MIC of MRB, 30 of MG alone, 100 µg/mL of MG alone and MG in combination with sub-MIC of MRB, respectively (Figures 5A and B).

To determine the mechanism underlying the adhesion and invasion inhibition, the expression level of *rck* gene, which is important for host cell invasion via the zipper mechanism, was evaluated via qRT-PCR. Accordingly, *rck* was downregulated by 26.7, 22.2 and 68.4% for bacteria treated with only sub-MIC of MRB, MG alone (30 µg/mL) and MG in combination with sub-MIC of MRB, respectively (Figure 5C).

**Figure 6:**
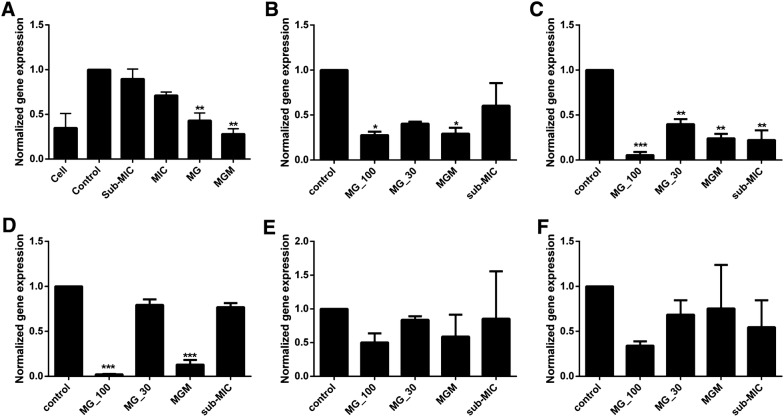
**Downregulation of** ***Salmonella*** **Typhimurium virulence genes by methyl gallate (MG). A** Effect of MG on *rac*-*1* expression, **B** effect of MG on *cheY* expression, **C** effect of MG on *ompD* expression, **D** effect of MG on *sipB* expression, **E** effect of MG on *lexA* expression, and **F** effect of MG on *ompF* expression. Bacteria were cultured with MG at different concentrations and incubated for 13 h in a non-shaking incubator aerobically at 37 °C. Gene expression levels were quantified via quantitative reverse transcription polymerase chain reaction. Control, inoculated with *Salmonella* Typhimurium but left untreated; MG-_100, inoculated with *Salmonella* Typhimurium and treated with 100 µg/mL of MG; MG_30, inoculated with *Salmonella* Typhimurium and treated with 30 µg/mL of MG; MGM, inoculated with *Salmonella* Typhimurium and treated with the combination of MG (30 µg/mL) and sub-MIC of MRB; Sub-MIC, inoculated with *Salmonella* Typhimurium and treated with sub-MIC of MRB. Values indicated are the mean ± SEM (*n* = 3; ***P-value < 0.001).

*Salmonella* invasion in epithelial cells was facilitated upon overexpression of Rac-1 and Cdc42 proteins. Hence, it is essential to determine the effect of MG on these Rho GTPases family members. Their gene expression levels were determined via the invasion assay. *rac*-*1* suppression by sub-MIC of MRB alone was 10.3%. Whereas, MG suppressed *rac*-*1* in Caco-2 cells by 56.9 and 71.9% alone and in combination with sub-MIC of MRB, respectively, which was significantly different (*P* = 0.0009) from that of the non-treated controls. However, no significant difference was observed in *cdc42* gene expression levels (data not shown) (Figure 6A).

In addition, the effect of MG on expression of *Salmonella* Typhimurium virulence genes essential for invasion and adhesion were assessed via qRT-PCR. The sub-MIC of MRB downregulated the expression of *cheY*, *ompD*, *sipB*, *lexA*, and *ompF* by 39.7, 77.8, 23.1, 45.4 and 14.6%, respectively. MG (30 µg/mL) downregulated *cheY*, *ompD*, *sipB*, *lexA*, and *ompF* by 59.6, 60.2, 20.5, 31.4, and 16.2%, respectively. Furthermore, the combination of the same concentration of MG with sub-MIC of MRB downregulated *cheY*, *ompD*, *sipB*, *lexA*, and *ompF* by 70.7, 76, 87, 24.6, and 41.1%, respectively. Gene expression was inhibited in a dose-dependent manner and differed significantly (*P* < 0.033) from the non-treated control groups, except for *lexA* and *ompF* genes (Figure 6).

## Discussion

*Salmonella* Typhimurium causes infections in humans and animals by adhering to host cells. Unlike other bacteria, *Salmonella* uses two different strategies to invade host cells [8]. Furthermore, drug resistance has emerged as a major public health concern. Currently, drug resistance has extended to the most conserved drugs including fluoroquinolones. *Salmonella* Typhimurium has developed resistance to MRB and enrofloxacin, which are widely used to treat multi-drug resistant bacteria in the veterinary medicine [15]. Hence, a substitute or combinatorial therapy is critical to limit the rate of infection rate and to reduce the occurrence of drug resistance. Therefore, in this study, we evaluated the antibacterial activity of MG and its mechanism underlying the inhibition of adhesion, invasion, and intracellular survival of *Salmonella* Typhimurium alone and in combination with MRB.

In this study, MG displayed antibacterial activity against field and laboratory strains (ATCC 14028) of *Salmonella* Typhimurium. The antibacterial activity of MG has also been reported previously [16, 23]. This finding is concurrent with reports on multidrug-resistant *Shigella* spp. and nalidixic acid resistant bacteria [18, 27]. However, no cytotoxicity and antibacterial activity of MG was observed at 30 µg/mL, showing that this concentration had no effect on mammalian cells and bacterial growth. Thus, we used this concentration to determine the effect of MG on the bacterial adhesion, invasion, and intracellular survival.

Pathogenic bacterial adhesion to host surfaces is an essential step in the pathogenesis of almost all infections, defining tissue tropism to specific surface receptors and resistance to physical elimination of extracellular fluids at mucosal sites [28]. Hence, adhesion plays a significant role in bacterial survival and replication. In this study, we reported that MG significantly reduced the adherence of *Salmonella* Typhimurium to host cell surfaces, which was further reduced in combination with the sub-MIC of MRB. Prevention of bacterial adhesion is a critical step to interfere with bacterial pathogenesis and colonization at the early phase of infection [29].

Once adhered to the host cell surface, bacteria penetrate cells via attachment with surface receptors or by direct translocation of bacterial proteins into the host cell. Invasion of *Salmonella* into host cells is critical for its survival and establishment of infection in the host. *Salmonella* Typhimurium is specialized in using the “trigger” and the “zipper” mechanisms to invade cells, using their specialized S*almonella* pathogenicity island-1 type III secretion system (SPI-1 T3SS) [7, 8]. Our findings indicate that MG can inhibit *Salmonella* invasion both in intestinal epithelial cells and macrophages when administered alone and in combination with MRB. This inhibitory effect is critical in reducing infection and diseases due to *Salmonella* Typhimurium.

MG inhibits bacterial invasion through various potential mechanisms. Bacterial motility is one of the phenomena that increases the rate of bacterial invasion [30]. Hence, inhibiting bacterial motility could reduce bacterial invasion. Our results indicated that motility of *Salmonella* Typhimurium was suppressed by MG alone and in combination with MRB. This reduction might probably result from the downregulation of the *cheY* gene by MG. CheY is a response regulator of the chemotaxis machinery, which regulates flagellar rotation of a motile bacterium including *Salmonella* [31]. CheY has high affinity for a switch component, FliM, at the flagellar motor, which alters flagellar rotation from the counterclockwise to the clockwise direction [32]. Suppression of cheY prevents directional switching of flagellar motors and renders the bacteria non-chemotactic, which further reduces bacterial adhesion and invasion. Furthermore, MG affects the proton motive force and ATP synthesis in Gram-negative bacteria, which is required for the synthesis and expression of motility- and other related genes [18].

Along with motility, penetration of *Salmonella* into the mucosal tissues of their hosts is mediated by various genes expressed from SPI-1 [33]. Furthermore, QS genes play a significant role in bacterial invasion of host cells. Hence, its inhibition reduces invasion of *Salmonella*, which in turn decrease its virulence [25]. In this study, we have shown that MG downregulated *Salmonella* Typhimurium QS genes (*sdiA* and *srgE*). *Salmonella* may perform QS for effective invasion under suitable conditions. In *Salmonella* Typhimurium, *sdiA* and *srgE* are regulated by the transcription factor LsrR. LsrR represses *InvF* expression and transcription of the InvF-regulated genes within SPI-1 and flagella genes. Hence, it impairs bacterial invasion of mammalian cells. In addition, when *Salmonella* approach a sufficient population level under a specific condition, QS might modulate the expression of virulence factors [34]. Concurrent with these suggestions, the inhibition of QS genes by MG and its combination with MRB could downregulate *Salmonella* virulence genes, which are specifically important for adhesion and invasion of cells.

MG suppressed *rck*, which is important in the receptor-mediating (zipper) mechanism of *Salmonella* invasion and entry into cells [7]. Downregulation of this gene reduces the invasion of *Salmonella* Typhimurium significantly. In *Escherichia coli*, overexpression of Rck enables the non-invasive bacterial adhere and invasion of fibroblastic cells [35]. The downregulation of *rck* might also be caused by the suppression of the *Salmonella* QS genes, specifically *sdiA* by MG. This might suggest that MG inhibits *Salmonella* invasion by blocking the zipper mechanism of invasion.

*rac*-*1*, an inducer of cytoskeletal remodeling [25], was also downregulated during treatment with MG after *Salmonella* Typhimurium invasion. The downregulation of *rac*-*1* genes of the host cell, whose activation is critical because it enhances the accumulation of actin filaments at sites of bacterial entry and a necessary step for bacterial invasion, is essential in inhibiting invasion. Rac-1 has also indicated to have a role in the “zipper” mechanism of invasion in uropathogenic *E. coli* [36, 37]. This result strongly supports our finding in that MG follows the same mechanism to inhibit invasion of *Salmonella* Typhimurium in the intestinal epithelial cells.

The inhibitory activity of MG alone or in combination with MRB was evident from the downregulation of *ompD*, a porin protein. Hence, the downregulation of *ompD* could be another factor for the reduction of adhesion and invasion of *Salmonella* Typhimurium in mammalian cells. This is concurrent with the findings of Ipinza et al. [38] who described the role of ompD in *Salmonella* invasion and intracellular survival in RAW264.7 cells and BALBc mice. The significance of ompD in adherence of *Salmonella* Typhimurium was also indicated in human monocytes and intestinal epithelial cells [39]. Suppression of the outer membrane protein genes *ompD* and *ompF* was associated with the disruption of the outer membrane of *Salmonella* Typhimurium by MG, which can probably affect both proton motive force and ATP synthesis, resulting in disorganizing the bacterial membrane [18].

*Salmonella* expresses SPI-1 virulence genes to invade host cells. SipB is one of the effector proteins of SPI-1 T3SS, which facilitates *Salmonella* entry into the host cell [40]. *sipB* was downregulated by MG in our study. Although it did not affect the growth of *Salmonella*, its suppression significantly decreased adherence, invasion, and virulence [41]. SipB also affects membrane fluidity and alters bacterial osmotolerance, thereby influencing membrane integrity [42]. This can potentially prevent *Salmonella* from penetrating host cells.

In conclusion, in this study, we have reported the in vitro effects of MG in preventing bacterial adherence and entrance and intracellular survival in the host cells, either alone or in combination with MRB, a widely used antibiotic in veterinary medicine. MG downregulated QS genes in *Salmonella* infections and suppressed the *rac*-*1* gene of the host cells. This may suggest that MG might interfere with the zipper mechanism of *Salmonella* Typhimurium to inhibit its invasion. MG also downregulated the virulence genes of *Salmonella* Typhimurium, which are critical for invasion of host cells. This activity of MG could demonstrate its ability to limit pathogenic bacterial infections and diseases of animals, specifically diseases caused by *Salmonella* Typhimurium. Furthermore, its effectiveness in combination with antimicrobials, such as MRB, against both susceptible and drug resistant bacterial strains revealed its paramount significance in combating antimicrobial resistance. However, further in vivo investigation on the clinical output of MG alone and in combination with other antibacterial agents is warranted.

## Additional file

**Additional file 1.** **Primers used for quantitative reverse transcription polymerase chain reaction.**

## Abbreviations

AHL: *N*-acyl homoserine lactone
ATP: adenosine triphosphate
CFU: colony-forming unit
LB: Luria Bertani
MG: methyl gallate
MIC: minimum inhibitory concentration
MOI: multiplicity of infection
MRB: marbofloxacin
QS: quorum sensing
SPI: *Salmonella* pathogenicity island
T3SS: type three section system
