# Supplementary material for: Inhibition of Salmonella Typhimurium adhesion, invasion, and intracellular survival via treatment with methyl gallate alone and in combination with marbofloxacin
Source: Vet Res. 2018 Oct 4;49:101. doi: 10.1186/s13567-018-0597-8 (PMC6389159; doi:10.1186/s13567-018-0597-8)
Supplement: Supplementary file 1 — Additional file 1. Primers used for quantitative reverse transcription polymerase chain reaction. [file 13567_2018_597_MOESM1_ESM.docx]

| Target gene | Primer sequence |
| --- | --- |
| *srgE* | 5′- GCGCAGGTTGGTATTACTTG-3′  5′- GGCAGATTGTTCATGATTGC-3′ |
| *sdiA* | 5′- TTACATTGGGATGACGTGCT-3′  5′- AACTGCTACGGGAGAACGAT-3′ |
| *iNOs* | 5′-CCCTTCCGAAGTTTCTGGCAGCAGC-3′  5′-GGCTGTCAGAGCCTCGTGGCTTTGG-3′ |
| *rrsG* | 5′-GTTACCCGCAGAAGAAGCAC-3′  5′- CACATCCGACTTGACAGACC 3′ |
| *sipB* | 5′-ACGCGCAAAGCCGAGGAAAC-3′  5′-CCCGTCGCCGCCTTCAC-3′ |
| *ompF* | 5′-CGTGCTGGCGGTTTGTTGAC-3′  5′-TTGCTGTACGCTGCGGTGAC-3′ |
| *cheY* | 5′-TTATCTCCGACTGGAACATGC-3′  5′-GACCATCAACACGGGTAACG-3′ |
| *ompD* | 5′-GCAACCGTACTGAAAGCCAGGG-3′  5′-GCCAAAGAAGTCAGTGTTACGGT-3′ |
| *sipB* | 5′-ACGCGCAAAGCCGAGGAAAC-3′  5′-CCCGTCGCCGCCTTCAC-3′ |
| *rck* | 5′-GTTGTATCCCGGCATGCTGAT-3′  5′-ATATGCCCAGAGCCGGATAGAG-3′ |
| *lexA* | 5′-TCGCGAGGTATCCGTCTG-3′  5′-TATGTACCGCCAGCAAATCC-3′ |
| *rac1* | 5′-CCTGATGCAGGCCATCAAG-3′  5′-AGTAGGGATATATTCTCCAGGAAATGC-3′ |
| *β-actin* | 5′- GGACTTCGAGCAGGAGATGG -3′  5′- GCACCGTGTTGGCGTAGAGG -3′ |
